# Evaluation of Transcriptomic Regulations behind Metabolic Syndrome in Obese and Lean Subjects

**DOI:** 10.3390/ijms21041455

**Published:** 2020-02-20

**Authors:** Magdalena Paczkowska-Abdulsalam, Magdalena Niemira, Agnieszka Bielska, Anna Szałkowska, Beata Anna Raczkowska, Sini Junttila, Attila Gyenesei, Edyta Adamska-Patruno, Katarzyna Maliszewska, Anna Citko, Łukasz Szczerbiński, Adam Krętowski

**Affiliations:** 1 Clinical Research Centre, Medical University of Białystok, M. Skłodowskiej-Curie 24A, 15-276 Białystok, Poland; 2 Department of Endocrinology, Diabetology and Internal Medicine, Medical University of Białystok, M. Skłodowskiej-Curie 24A, 15-276 Białystok, Poland; 3 Vienna Biocenter Core Facilities, Dr.-Bohr-Gasse 3, 1030 Vienna, Austria

**Keywords:** transcriptomics, obesity, obesity phenotypes, metabolic syndrome, cardiovascular disease

## Abstract

Multiple mechanisms have been suggested to confer to the pathophysiology of metabolic syndrome (MetS), however despite great interest from the scientific community, the exact contribution of each of MetS risk factors still remains unclear. The present study aimed to investigate molecular signatures in peripheral blood of individuals affected by MetS and different degrees of obesity. Metabolic health of 1204 individuals from 1000PLUS cohort was assessed, and 32 subjects were recruited to four study groups: MetS lean, MetS obese, “healthy obese”, and healthy lean. Whole-blood transcriptome next generation sequencing with functional data analysis were carried out. MetS obese and MetS lean study participants showed the upregulation of genes involved in inflammation and coagulation processes: granulocyte adhesion and diapedesis (*p* < 0.0001, *p* = 0.0063), prothrombin activation pathway (*p* = 0.0032, *p* = 0.0091), coagulation system (*p* = 0.0010, *p* = 0.0155). The results for “healthy obese” indicate enrichment in molecules associated with protein synthesis (*p* < 0.0001), mitochondrial dysfunction (*p* < 0.0001), and oxidative phosphorylation (*p* < 0.0001). Our results suggest that MetS is related to the state of inflammation and vascular system changes independent of excess body weight. Furthermore, “healthy obese”, despite not fulfilling the criteria for MetS diagnosis, seems to display an intermediate state with a lower degree of metabolic abnormalities, before they proceed to a full blown MetS.

## 1. Introduction

Metabolic syndrome (MetS), with its growing worldwide prevalence and significant impact on the risk of cardiovascular disease and type 2 diabetes development, has emerged as a serious public health concern. The components of MetS include abdominal obesity, hypertension, disturbed glucose metabolism, elevated triglycerides, and decreased HDL cholesterol. Individuals affected by the syndrome concurrently present with pro-inflammatory and prothrombotic states. Despite most organizations recognizing MetS as a cluster of factors increasing the risk of type 2 diabetes and cardiovascular disorders, the subject of a single precise definition has been under debate for years. Several sets of criteria have been proposed to date, mostly differing in how each of the components is detected clinically, and which one of them is obligate to diagnose dysmetabolic phenotype. In 2009, a joint interim statement was released with a single set of components and their cut points, except for waist circumference, for which ethnic-specific cut-offs are recommended [1]. The harmonized definition became the most commonly accepted with any three of five factors required: elevated waist circumference, elevated triglycerides, reduced HDL cholesterol, elevated blood pressure, and elevated fasting glucose.

The complexity of MetS is further confirmed by the lack of consensus in the research community regarding the role of factors responsible for its development. Multiple mechanisms have been suggested to confer to MetS pathophysiology, with insulin resistance and disturbances in lipid metabolism believed to be major contributors [2,3]. It has also been associated with oxidative stress [4] and mitochondrial impairment [5]. Some investigators regard excessive adipose tissue, accumulating as a consequence of a positive calorie intake balance and sedentary lifestyle, to be a key player in the causation of MetS [6]. This view is in agreement with observed obesity epidemic, coupled with rising MetS prevalence. In spite of substantial evidence supporting the concept of obesity being an important factor in the development of other components of MetS, some researchers suggest the disproportion between calorie intake and energy expenditure to trigger mechanisms behind MetS in the first place, before the excessive adipose tissue starts to contribute [7]. Few studies reported that calorie restriction reverses the majority of metabolic risk factors even though the continuing obesity was present [8,9]. 

In spite of a great interest from the scientific community and numerous studies being conducted, there is still no agreement on the exact contribution of each of MetS risk factors to its pathophysiology. The purpose of the study was, thus, to evaluate molecular signatures in peripheral blood of individuals affected by MetS and/or obesity through a whole-blood transcriptome analysis with the use of NGS technology. We intended to closely investigate “healthy obese” phenotype in order to define the role of obesity in the development of metabolic abnormalities as well as to assess the extent of the “healthy obese” exposure to metabolic and cardiovascular complications. Although such an approach required multiple comparisons and recruitment of several different study groups, it allowed for a comprehensive assessment of contribution of each of the factors, obesity and MetS, to metabolic health deterioration.

## 2. Results

### 2.1. Study Group Characteristics

The characteristics of subjects that participated in the study are shown in Table 1. Participants were recruited to four different groups depending on their BMI and metabolic health status, which was assessed with the harmonized MetS definition. Then, 1204 individuals underwent physical examination, however the majority of them met the exclusion criteria. Eventually, each of the groups was comprised of eight individuals, all matched for gender and age. There were no differences in the major leukocyte types between the study groups. Obese individuals affected by MetS showed higher waist circumference, BMI, triglycerides, fasting plasma glucose, and lower HDL-C than the subjects in all other groups. MetS obese presented significantly higher fasting plasma glucose levels and diastolic blood pressure when compared to healthy obese study participants. Body composition analysis revealed higher body fat percentage and a higher amount of both visceral and subcutaneous adipose tissue in MetS obese than in healthy obese individuals. The percentage of subcutaneous adipose tissue, though, showed to be lower in MetS obese than in healthy obese subjects. When comparing MetS lean to healthy lean individuals, those affected by MetS had significantly higher body fat percentage, despite no differences in BMI between both groups. Healthy obese study participants presented as insulin resistant with significant differences in HOMA-IR values, when compared to healthy lean individuals.

### 2.2. Data Analysis

Differential expression analysis identified groups of genes with condition-dependent behavior. Table 2 presents the number of up- and down-regulated genes after stringent statistical testing and subsequent filtering of the results by fold change and P value. In the next step, the dataset for each comparison was submitted to Ingenuity Pathway Analysis (IPA) software, which revealed the enrichment in canonical pathways, and disease and function annotations. The results are presented below, separately for each comparison.

#### 2.2.1. Metabolic Syndrome (MetS) Obese vs. “Healthy Obese”

According to the IPA analysis, sequencing results for MetS obese showed significant enrichment in canonical pathways related to inflammation and coagulation: “Granulocyte/Agranulocyte Adhesion and Diapedesis”, “Coagulation System”, “Intrinsic Prothrombin Activation Pathway”, “Integrin Signaling”. Few disease-specific pathways were also listed, including “Hepatic Fibrosis” and “Atherosclerosis Signaling” (Table 3). The analysis of diseases and functions showed enrichment in processes associated with inflammatory disease (severe inflammatory disorder, chronic inflammatory disorder), cell-to-cell signaling and interaction (aggregation and binding of cells), hematological system development and function (aggregation of blood cells, activation of blood platelets, coagulation of blood), and organismal injury (arteriosclerosis).

#### 2.2.2. MetS Lean vs. Healthy Lean

MetS lean individuals showed the upregulation of molecules associated with pro-inflammatory and pro-thrombotic states: “Granulocyte/Agranulocyte Adhesion and Diapedesis”, “Coagulation System”, “Extrinsic/Intrinsic Prothrombin Activation Pathway” (Table 4). Similarly, IPA disease or function annotations included activation, aggregation and binding of blood platelets, adhesion of vascular endothelial cells, and formation of blood clots.

#### 2.2.3. “Healthy Obese” vs. Healthy Lean

The results of IPA analysis indicate the upregulation of genes within pathways involved in protein synthesis, cellular growth and development, including “EIF2 Signaling”, “Regulation of eIF4 and p70S6K Signaling” and “mTOR Signaling” (Table 5). Majority of genes belonging to “Mitochondrial Dysfunction” and “Oxidative Phosphorylation” canonical pathways were also observed to be up-regulated in “healthy obese” subjects, when compared to the healthy lean. Ingenuity diseases and functions enriched in the dataset show the up-regulation of genes involved in processes related to renal disease (chronic kidney disease), CVDs (peripheral arterial/vascular disease), and mitochondrial deficiencies.

## 3. Discussion

The proposed study is the first to perform a detailed gene expression profiling with the use of next generation sequencing technology to assess the differences in molecular mechanisms in peripheral blood of patients with MetS depending on their degree of obesity. 

Peripheral blood is an easily accessible material with a very low risk associated with its collection, in contrast to invasive tissue biopsies. Blood interacts with every organ in the body and plays a key role in inflammation, immunity, and physiological homeostasis. Molecular profiles of circulating blood were suggested to reflect pathological events throughout the organism. Growing evidence demonstrates the existence of gene expression signatures characteristic for certain disease phenotypes or environmental influences [10,11,12,13]. Moreover, researchers compared the peripheral blood transcriptome to gene expression patterns in nine different human tissues, demonstrating that more than 80% of expressed genes are shared between blood and any of the tissues [14]. All these findings suggest that circulating blood may serve as a reliable source of diagnostic biomarkers and potentially provide the insight into molecular processes behind the condition under investigation, which is further confirmed by the presented research. On the other hand, though, one of the key challenges of the analysis of whole blood transcriptome is the overabundance of globin mRNA, which may decrease the ability to detect transcripts with low expression levels. Several experimental protocols of globin depletion have been proposed, which however significantly reduce the amount of extracted RNA following depletion [15] and compromise the quality of the nucleic acid [16]. Any loss of RNA caused by depletion might affect the sequencing data, particularly for samples with a limited amount of starting material. Increasing the sequencing depth of coverage was suggested as a solution that replicates the effect of experimental globin depletion [15] and thus was applied in this study. Another considerable issue associated with the use of peripheral blood of patients in gene expression studies is RNA stability. Studies report that RNA undergoes degradation already during collection and storage, thus affecting the results of transcriptome analysis. Any additional procedure following sample acquisition, such as peripheral blood cells preparation, involves a time delay before RNA stabilization and therefore may expose blood cells to factors that trigger ex vivo changes in gene expression patterns, not related to the condition under study [17,18]. The Food and Drug Administration addressed the problem of blood transcriptome profiling standardization and approved the PAXgene system for whole blood collection and RNA stabilization. In order to preserve high RNA quality and unchanged gene expression profile, the presented study involved samples collected and stored using the PAXgene system.

RNA-seq was chosen as a method for transcriptome profiling in order to capture the whole-genome gene expression levels. In contrast to targeted approaches such as real-time PCR, NGS provides the information about all transcripts present in the sample and does not require any a priori knowledge about gene expression patterns or transcript nucleotide sequences. Although the costs of sequencing have decreased significantly since the first sequencers entered the market, the analyses are still highly priced when compared to RNA-seq’s biggest competitor, i.e., microarray technology. The challenging storage of huge amounts of data produced during sequencing runs and a very complex bioinformatic analysis are two other major barriers next to the high cost of the sequencing that refrain most of the researchers from choosing NGS as a method of transcriptome analysis. However, it was demonstrated that RNA-seq detects more differentially expressed genes when compared to microarrays [19,20]. Moreover, the method is reported to be more sensitive in detecting low abundance transcripts and genes with very high expression [21]. Unlike microarrays, it also avoids technical issues associated with cross-hybridization, non-specific hybridization, or limited detection range for some probes [19]. Taking all mentioned benefits into consideration, RNA-seq appears to be a superior method for transcriptome analysis, and therefore was applied in this project to study MetS patients. Regarding computational analysis of transcriptomic data, we decided to focus on overrepresented biological processes, functions, and pathways rather than on identifying individual genes, therefore traditional P values were used to investigate molecules’ enrichment. An applied approach is consistent with the work of other researchers, who investigated the skeletal muscle gene expression profile in MetS patients with the use of microarrays [22]. The analysis of pathway enrichment, in contrast to a single gene approach, yields the information on the network of biological interactions, allowing to detect changes at a higher biological level than individual genes or molecules, and thus contributing to a better understanding of the complexity of the disease [23]. It also seems a reasonable choice, when taking into account the limited statistical power of the study, resulting from low sample size, and the nature of investigated condition. It should be noted that the recruitment of study participants posed a significant challenge. Out of more than 1200 individuals, only 32 were eligible to take part in the study. A strong association between age and metabolic health status was observed. Despite huge efforts from our side to match individuals between groups with as many parameters as possible, the definition of MetS remains extremely broad, and subjects, identified as affected by MetS, turned out to compose a quite heterogeneous group. Future studies should not only aim at a significantly larger sample size but also consider dividing MetS patients into subgroups, depending on which MetS definition criteria they fulfill. In addition, our study focuses on gene expression only, which does not always reflect protein levels. The analysis of proteome could yield additional information, allowing the studying of mechanisms behind MetS in a more comprehensive way.

The enrichment analysis of data coming from samples of MetS study participants identified a significant group of overrepresented pathways, related to the processes of inflammation, coagulation, and thrombosis. Even though none of the pro-inflammatory markers are listed among MetS diagnosis criteria, the link between these conditions has been confirmed by several studies [24,25]. Obesity, as well as excessive nutrient intake, have been observed to contribute to the onset of inflammatory state [26,27,28]. In addition to enhanced secretion of inflammatory cytokines, abnormalities related to procoagulant factors were also noted in patients with MetS [29,30]. The activation of pro-inflammatory and pro-thrombotic pathways, resulting in the onset of chronic inflammatory process in MetS obese subjects, most probably affects the vascular system, causing the accumulation of fatty materials such as cholesterol, subsequent artery walls thickening, and finally, the development of atherosclerosis. Excess body weight is considered to be a major contributor to the activation of inflammatory and coagulation processes in MetS patients, and the upregulation of these pathways was observed in MetS obese when compared to “healthy obese” study participants. The results for the group of lean individuals, however, suggest that MetS is related to the process of inflammation and thrombosis, regardless of obesity. Obesity itself may act as a factor increasing the severity of the condition, so the effects of obesity-induced and MetS-induced inflammation and thrombosis appears to add up in MetS obese patients.

The analysis of differentially expressed genes between “healthy obese” and healthy lean study participants revealed a significant up-regulation of pathways associated with protein synthesis, including mTOR signaling pathway. Interestingly, growing evidence suggests that increased mTOR signaling leads to alterations of cellular metabolic signaling, which in turn contributes to the development of insulin resistance and obesity-related diseases, including diabetes, cardiovascular disorders, and cancer [31]. mTOR signaling pathway has been reported to be frequently activated in various tissues during conditions of excessive nutrient intake [32]. It has been noted that chronic activation of mTORC1, one of the mTOR protein complexes, contributes to obesity by enhancing excess fat accumulation in white adipose tissue, liver and muscle, which promotes insulin resistance. This, in turn, leads to a reduced glucose uptake and glycogen synthesis in the liver and muscle, and increased gluconeogenesis and glucose release by liver, further exacerbating hyperglycemia and hyperinsulinemia generated by excess nutrients [33]. Additionally, persistent activation of mTORC1-S6K1 signaling leads to pancreatic β-cell apoptosis through phosphorylation and subsequent proteosomal degradation of insulin receptor substrate 2. It has been suggested as a contributing mechanism as to how β-cell mass is decreased by chronic hyperglycemia in the pathogenesis of type 2 diabetes [34]. mTORC1 activity is enhanced by elevated levels of circulating pro-inflammatory cytokines, insulin, and nutrients in obese animals. It not only contributes to the adipose tissue expansion through the activation of lipogenic factors, but also promotes insulin resistance in adipose tissue through S6K1-mediated inhibition of insulin signaling [35]. Reduced activity of insulin in adipose tissue likely exacerbates systemic insulin resistance by promoting free fatty acids release by adipocytes, ectopic fat deposition, and lipotoxicity [36]. Enhanced protein synthesis triggered by mTORC1 activation may also induce insulin resistance by promoting ER stress and the unfolded protein response (UPR) [36,37]. Activated mTORC1 inhibits insulin signaling in muscles of obese rodents fed a high-fat diet, which in turn reduces glucose uptake by the muscle, giving rise to systemic insulin resistance [36]. Interestingly, conditions associated with increased mTORC1 activity, such as high fat diet, obesity, and type 2 diabetes, all impair mitochondrial biogenesis and function in muscles. Similarly to muscle and adipose tissue, mTORC1 activity was observed to be increased in the livers of obese rodents, resulting in the degradation of insulin receptor substrate 1 and the onset of hepatic insulin resistance [36]. A recent study by Uchinaka et al. reported that the activation of mTOR signaling contributes to MetS pathophysiology and associated complications [38].

The majority of identified genes belonging to pathways related to oxidative phosphorylation and mitochondrial dysfunction showed higher expression in “healthy obese” compared to healthy lean individuals. The finding is consistent with the study by Gosh et al., who investigated peripheral blood gene expression levels in obese subjects and demonstrated the up-regulation of oxidative phosphorylation pathways [39]. Oxidative phosphorylation is a process of ATP production using energy derived from the transfer of electrons, which takes place within mitochondria, the major energy source for most cells [40]. When the structure and function of mitochondria remains intact, the production of ROS is modest. In the case of mitochondrial dysfunction, ROS-mediated oxidative stress overpowers the antioxidant defense system. The up-regulation of both pathways within differentially expressed genes in our dataset may indicate increased energy demands or a substantial increase in ROS generation in “healthy obese” individuals. Several studies reported a crucial role of oxidative stress in the pathogenesis of cardiovascular disorders and type 2 diabetes [41]. It has been also suggested as a secondary mechanism triggering inflammatory response in obese individuals [42] and most importantly an early initiator of MetS [43]. All things considered, the results of our study largely question the existence of a “healthy obese” phenotype. Even though “healthy obese” individuals do not fulfill the criteria for MetS diagnosis, they seem to be just one step behind the obese with a full-blown MetS. 

To conclude, the analysis of a whole blood transcriptome turned out to be a potentially useful tool for the assessment of metabolic health status. It revealed groups of genes with condition-dependent behavior, providing a specific signature for each of the phenotypes under study. Peripheral blood, thus, seems to reflect changes in biological processes in other tissues of human body. Moreover, MetS appears to be related to mechanisms leading to the state of inflammation and changes in the vascular system, independently of excess body weight. Finally, our results indicate that although “healthy obese” do not fulfill the criteria for MetS diagnosis, they already seem to be affected by the disorder on a molecular level. “Healthy obesity” could be then an intermediate state, with individuals presenting a lower degree of metabolic abnormalities before they proceed to a full blown MetS.

## 4. Materials and Methods

### 4.1. Ethical Statement

The study protocol applied in present research project received approval of the local Ethic Committee (R-I-002/35/2009 on January 29,2009; R-I-002/233/2015 on 28 May 2015) and complies with good clinical practice guidelines. Written consent was obtained from all study participants.

### 4.2. Study Population

A population-based sample from the 1000PLUS cohort was enrolled in the present study, recruited between 2007–2017 as described previously [44,45,46]. The study is registered at ClinicalTrials.gov as NCT03792685. Individuals were recruited to four groups: lean with metabolic syndrome (MetS lean), obese with metabolic syndrome (MetS obese), “healthy obese”, and healthy lean, matched for gender and age. Eventually, each of the groups under investigation was comprised of eight subjects. Metabolic syndrome (MetS) was diagnosed in accordance with the harmonized definition established by IDF, AHA, and NHLBI. Individuals were considered as affected by MetS when presenting any 3 of the 5 factors: central obesity (waist circumference ≥ 94 cm in men and ≥ 80 cm in women), high triglycerides (≥ 150 mg/dL), reduced HDL-C (< 40 mg/dL in men, < 50 mg/dL in women), elevated blood pressure (≥ 130/85 mmHg), or documented use of antihypertensive therapy, and high fasting glucose (≥ 100 mg/dL) [1]. None of the subjects enrolled in the study were smokers. Out of all study participants, three of them (one in each of the groups: MetS obese, MetS lean, healthy lean) were treated with ACE inhibitors only (perindopril). Apart from metabolic health status, BMI as a measure of obesity was also taken into account. In order to classify individuals as normal weight or obese, thresholds proposed by WHO were used. BMI was defined as weight in kilograms divided by the square of height in meters. Study participants with BMI below 25 were classified as normal weight, subjects with BMI of 30 or more were considered obese.

### 4.3. Clinical Parameters

Physical examination and collection of blood samples from study participants were both conducted on the same day. Anthropometric measurements were made, including height, weight, waist circumference, and blood pressure. Bioelectrical impedance analysis was performed using Maltron BioScan 920-2 (Maltron International Ltd., Essex, UK) in order to estimate body fat percentage and the amounts of visceral and subcutaneous adipose tissue. Biochemical blood variables were also evaluated by the Medical Laboratory at the Clinical Research Centre. The value of HOMA-IR was calculated according to the formula: fasting insulin (μU/mL) × fasting glucose (mg/dL)/405. 

### 4.4. Sample Collection and RNA Extraction

Blood samples required for RNA extraction were collected into PAXGene Blood RNA tubes (Qiagen, Hilden, Germany) using BD Vacutainer Safety-Lok blood collection set (Becton Dickinson, Franklin Lakes, NJ, USA) and incubated 2 h at room temperature after being inverted 8 to 10 times. The tubes were further stored in −20 °C for 24 h and then moved to −80 °C until needed. PAXGene Blood RNA tube was always the last tube drawn during phlebotomy procedure.

Total RNA was purified from 2.5 mL of human whole blood drawn to PAXgene Blood RNA Tube (Qiagen, Hilden, Germany) with the use of PaxGene Blood RNA Kit (Qiagen, Hilden, Germany) according to manufacturer’s instructions. The quality of isolated nucleic acid was examined with Agilent 2200 TapeStation instrument (Agilent Technologies, Santa Clara, CA, USA). Fluorescence-based quantitation method was applied to determine the concentration of extracted RNA, which was measured with the use of Qubit RNA BR Assay and Qubit 3.0 fluorometer (Thermo Fisher Scientific, Waltham, MA, USA).

### 4.5. NGS Library Construction and Sequencing

Libraries were constructed using TruSeq Stranded mRNA Library Prep Kit (Illumina, San Diego, CA, USA) following manufacturer’s instructions (part# 15031047 Rev. E). DNA libraries were further quantified using Qubit 3.0 fluorometer and Qubit dsDNA BR Assay Kit (Thermo Fisher Scientific, Waltham, MA, USA). Quality control analysis assessing size distribution and purity of each library was conducted with 2200 TapeStation Instrument and D1000 reagents (Agilent Technologies, Santa Clara, CA, USA). Indexed libraries were further normalized using Tris-HCl pH 8.5 with 0.1% Tween to achieve the concentration of 10 nM, and then pooled in equal volumes. Before sequencing, libraries were denatured and diluted to the concentration of 1.8 pM. A sequencing control library, derived from bacteriophage PhiX genome, was added as a 1% spike-in to each sequencing run to provide an in-run control and balanced fluorescent signals at each cycle improving the overall run quality. A 76-cycle paired-end sequencing runs were conducted on the NextSeq500 platform (Illumina, San Diego, CA, USA) loaded with NCS software (version 1.4). In each sequencing run, a pool of samples was sequenced on four lanes of the flowcell. Raw data in the form of base call files were demultiplexed and converted to fastq files upon completion of each run using BaseSpace Onsite v2.0 (Illumina, San Diego, CA, USA). Sequencing Analysis Viewer v1.11 software (Illumina, San Diego, CA, USA) was used to determine the quality of each sequencing run. RNA-seq data were deposited in the Gene Expression Omnibus (GEO) database (accession number: GSE145412).

### 4.6. Data Preprocessing and Normalization

The reads obtained from the sequencer were aligned against the Homo sapiens reference genome (Ensembl GRCh37 release) with STAR version 2.5.1b using the 2-pass alignment mode. HTSeq-count analysis was performed to calculate the percentage of reads within different genomic regions [47]. After alignment reads were annotated based on information derived from University of California, Santa Cruz (UCSC) Genome Browser [48]. The number of reads within each gene was counted using HTSeq tool version 0.5.4p3 [47]. The counts were further normalized with the TMM normalization method of the edgeR R/Bioconductor package (R version 3.2.0, Bioconductor version 3.1, Buffalo, NY, USA). For statistical testing, data were log transformed using the voom approach in the R Limma package.

### 4.7. Differential Expression Analysis

Following comparisons were performed to detect deferentially expressed genes between study groups:

- MetS obese vs. “healthy obese”
- MetS lean vs. healthy lean
- “Healthy obese” vs. healthy lean

Before statistical testing, genes with less than 20 reads across all samples were filtered out. Statistical testing was performed using Limma, which applies linear modelling with a modified t-test to calculate P values and fold changes. In the next step, filtering was performed in order to list genes that show the strongest evidence for being differentially expressed between compared groups. Fold change of 1.5 and *p* value of 0.05 were used as filtering criteria for each comparison. 

### 4.8. Pathway Enrichment Analysis

The lists of differentially expressed genes were submitted to Ingenuity Pathway Analysis (IPA) software (spring release 2017, Qiagen, Hilden, Germany). Each gene symbol was mapped to its corresponding gene object in the Ingenuity Knowledge Base. IPA pathway analysis identified canonical pathways, diseases, and functions overrepresented in each dataset. The significance of the overlap between experimental dataset and Ingenuity canonical pathway was determined based on a *p* value calculated using a right-tailed Fisher’s exact test.

## Figures and Tables

**Table 1:** Clinical characteristics of study participants.

Feature	Group 1 MetS Obese (*n* = 8)	Group 2 “Healthy Obese” (*n* = 8)	Group 3 MetS Lean (*n* = 8)	Group 4 Healthy Lean (*n* = 8)	1 vs. 2 *p* Value	3 vs. 4 *p* Value	2 vs. 4 *p* Value
Age (years)	47 ± 3	49 ± 7	49 ± 13	46 ± 4	0.8336	0.4005	0.4005
Waist circumference (cm)	121.50 ± 15.47	105.38 ± 15.47	85.13 ± 4.55	79.50 ± 8.11	0.0134	0.2434	0.0008
Triglycerides (mg/dl)	115.63 ± 25.59	100.13 ± 51.66	89.38 ± 39.54	68.88 ± 37.35	0.1722	0.2076	0.1275
HDL-C (mg/dl)	51.00 ± 13.98	53.00 ± 6.72	62.00 ± 23.11	64.13 ± 12.08	0.8335	0.4942	0.0736
Fasting plasma glucose (mg/dl)	106.75 ± 6.61	94.75 ± 4.65	105.38 ± 8.99	96.63 ± 6.19	0.0008	0.0582	0.5965
Systolic blood pressure (mmHg)	126 ± 16	124 ± 13	136 ± 13	113 ± 17	0.9581	0.0157	0.1715
Diastolic blood pressure (mmHg)	91 ± 7	81 ± 11	89 ± 9	79 ± 10	0.0262	0.1144	0.5990
HOMA-IR	4.87 ± 1.76	3.42 ± 0.88	2.66 ± 0.85	1.87 ± 0.79	0.0742	0.0587	0.0157
HOMA-β	155.12 ± 56.85	174.14 ± 74.51	89.10 ± 30.41	84.45 ± 32.48	0.7527	0.5995	0.0087
BMI (kg/m^2^)	39.79 ± 5.49	33.65 ± 2.40	24.09 ± 0.89	23.06 ± 1.47	0.0117	0.1409	0.0008
Body fat (%)	48.24 ± 6.65	40.31 ± 7.24	32.81 ± 3.15	28.98 ± 3.50	0.0587	0.0460	0.0063
VAT (cm^3^)	261.75 ± 90.55	180.88 ± 57.04	92.88 ± 26.87	67.75 ± 29.97	0.0742	0.1149	0.0008
SAT (cm^3^)	417.25 ± 117.19	378.63 ± 70.87	298.13 ± 56.38	244.00 ± 51.97	0.4945	0.0740	0.0023
VAT (%)	38.34 ± 8.24	32.03 ± 5.80	23.43 ± 3.73	21.00 ± 5.42	0.0929	0.4008	0.0033
SAT (%)	61.66 ± 8.24	67.97 ± 5.80	76.58 ± 3.73	79.00 ± 5.42	0.0929	0.4008	0.0033
VAT/SAT	0.63 ± 0.22	0.48 ± 0.13	0.31 ± 0.06	0.27 ± 0.09	0.1152	0.4008	0.0033

Values are means ± SD, statistical significance was calculated using the Mann–Whitney U-test. MetS, metabolic syndrome; HDL-C, high-density lipoprotein cholesterol; HOMA-IR, homeostasis model-assessment of insulin resistance; HOMA-β, homeostasis model assessment of β-cell function; BMI, body mass index; VAT, visceral adipose tissue; SAT, subcutaneous adipose tissue.

**Table 2:** Filtering summary for all comparisons.

Comparison	Genes with Altered Expression	Up-Regulated Genes	Down-Regulated Genes
**MetS obese vs. “healthy obese”**	902	440	462
**MetS lean vs. healthy lean**	973	482	491
**“healthy obese” vs. healthy lean**	808	542	266

**Table 3:** Enrichment in Ingenuity Canonical Pathways and Diseases or Functions Annotations among genes differentially expressed between MetS obese and “healthy obese” individuals, where the majority of genes within each pathway are up-regulated. Down-regulated genes are underlined.

MetS Obese vs. “Healthy Obese”			
**Ingenuity Canonical Pathways**		**Molecules**	***p*** ** Value**
Granulocyte Adhesion and Diapedesis		SELP, IL1B, MMP1, CXCL16, CLDN23, CLDN5, ITGB3, HRH2, PF4, CXCL5, FPR3, CXCL9, HRH1, CDH5, TNFRSF11B	<0.0001
γ-glutamyl Cycle		OPLAH, CHAC1, GGT5, ANPEP	0.0004
Agranulocyte Adhesion and Diapedesis		MYL9, CXCL16, SELP, PF4, IL1B, CXCL5, MMP1, CLDN5, CLDN23, HRH1, CDH5, CXCL9	0.0006
Glioma Invasiveness Signaling		DIRAS3, FGFR2, PLAU, ITGB5, TIMP2, ITGB3, RND2	0.0009
Coagulation System		F8, PROS1, F13A1, SERPINA1, PLAU	0.0010
Hepatic Fibrosis / Hepatic Stellate Cell Activation		MYL9, PDGFA, IL1B, COL10A1, EGF, FGFR2, COL20A1, MMP1, TIMP2, CXCL9, TNFRSF11B	0.0025
Intrinsic Prothrombin Activation Pathway		F8, PROS1, F13A1, COL10A1	0.0032
Atherosclerosis Signaling		SELP, PDGFA, IL1B, COL10A1, SERPINA1, ALOX12, MMP1, CLU	0.0066
Integrin Signaling		MYL9, ITGA2B, DIRAS3, ZYX, FGFR2, MYLK, CTTN, ITGB5, ITGB3, RND2, TTN	0.0083
**Ingenuity Disease or Function Annotation**		**Molecules**	***p*** ** Value**
Inflammatory Disease	severe inflammatory disorder	ACSL1, ANPEP, CLU, FGFR2, HIST1H2BD, HIST1H2BK, HP, ITGA2B, PDE5A, PGLYRP1, PTGS1, PYGL, SERPINA1, SLPI, SPARC, TIMP2, WNT11, ZYX, WNT9A	<0.0001
	chronic inflammatory disorder	ACSL1, ADM, ALOX12, ALPL, C3orf52, C4A/C4B, CLEC1B, CLU, COL10A1, CR1, CXCL5, CYP19A1, EGF, FGFR2, GP1BA, GSDMC, HP, HRH2, IL1B, ITGB3, KCND3, MGAM, MGLL, MMP1, PDE5A, PI3, PTGS1, RGS6, SELP, SLPI, SOCS3, TLR5, TRIM40, TRPM8, TUBA1A, TUBB1, WNT11, AHI1, BSN, CXCL9, EIF3E, FLT3LG, HRH1, IL23A, NKD1, POMC, RGS1, RPL11, RPL31, RPS24, TNFRSF11B, VSIG4, WDR78, WNT9A	0.0003
Cell-To-Cell Signaling and Interaction	binding of blood cells	C4A/C4B, CR1, GP1BA, GP6, IL1B, ITGA2B, ITGB3, PF4, PLAU, PTX3, SELP, SERPINA1, SIGLEC5, TLR5, ULBP2, CD209, CXCL9, RGS1, RLN2	<0.0001
Hematological System Development and Function	aggregation of blood cells	ALOX12, CLEC1B, F2RL3, GP1BA, GP6, ITGA2B, ITGB3, MYL9, PEAR1, PTX3, SELP, TIMP2, CD209, CXCL9	<0.0001
	activation of blood platelets	CLEC1B, GP1BA, GP6, ITGB3, PDGFA, PF4, SELP	<0.0001
	coagulation of blood	CLEC1B, F2RL3, GP1BA, GP6, ITGB3, PDGFA, PF4, PROS1, SELP, C4BPB	<0.0001
Organismal Injury and Abnormalities	arteriosclerosis	CA4, CACNA1E, CDR2L, CLU, EGF, HCAR2, HCAR3, HRH2, IL1B, ITGA2B, ITGB3, MGAM, PDE5A, PF4, PLAU, PTGS1, SCN1B, SELP, SOCS3, SPARC, TIMP2, TMEM163, TUBA1A, TUBB1, VEPH1, RGS1, TNFRSF11B, VSIG4	<0.0001

**Table 4:** Enrichment in Ingenuity Canonical Pathways and Diseases or Functions Annotations among genes differentially expressed between MetS lean and healthy lean individuals, where the majority of genes within each pathway are up-regulated. Down-regulated genes are underlined.

MetS Lean vs. Healthy Lean			
**Ingenuity Canonical Pathways**		**Molecules**	***p*** ** Value**
Granulocyte Adhesion and Diapedesis		CLDN5, SELP, PPBP, PF4, CCL22, MMP17, ITGB3, IL1A, CCL2, HRH4, ITGA4	0.0063
Extrinsic Prothrombin Activation Pathway		F12, PROS1, F2	0.0091
Agranulocyte Adhesion and Diapedesis		MYL9, CLDN5, SELP, MYL6, PPBP, PF4, CCL22, MMP17, IL1A, CCL2, ITGA4	0.0095
Coagulation System		F12, PROS1, F2, F2R	0.0155
Intrinsic Prothrombin Activation Pathway		F12, PROS1, F2	0.0417
Caveolar-mediated Endocytosis Signaling		ITGA2B, ITGB5, ITGB3, DYRK3, ITGA4	0.0468
**Ingenuity Disease or Function Annotation**		**Molecules**	***p*** ** Value**
Organismal Injury and Abnormalities	blood clot	ALOX12, BAAT, EPO, F12, F2, ITGA2B, ITGB3, PDE5A, PF4, PROS1, SELP, THBS1, F2R, PDE3B	0.0001
	thrombus	ALOX12, BAAT, EPO, F2, ITGA2B, ITGB3, PDE5A, PROS1, SELP, THBS1, F2R, PDE3B	0.0005
Inflammatory Response	activation of blood platelets	F2, GP6, ITGB3, PF4, SELP, THBS1, F2R	<0.0001
	aggregation of blood platelets	ALOX12, F12, F2, F2RL3, GP6, HBB, ITGA2B, ITGB3, MYL9, PRKG1, THBS1, F2R, RASGRP1	<0.0001
	binding of blood platelets	CCL22, F12, F2, GP6, ITGA2B, ITGB3, SELP	0.0002
Cardiovascular System Development and Function	adhesion of vascular endothelial cells	EGFL7, F2, ITGB3, PF4, PPBP, SELP, SPARC, THBS1, CCL2, F2R, IL1A, ITGA4, RICTOR, STAT1	<0.0001

**Table 5:** Enrichment in Ingenuity Canonical Pathways and Diseases or Functions Annotations among genes differentially expressed between “healthy obese” and healthy lean individuals, where the majority of genes within each pathway are up-regulated. Down-regulated genes are underlined.

“Healthy Obese” vs. Healthy Lean			
**Ingenuity Canonical Pathways**		**Molecules**	***p*** ** Value**
EIF2 Signaling		RPL24, RPL11, RPS3A, RPS27, RPL22L1, RPL39, RPL26, RPL35A, RPL7, RPS7, RPL35, RPS20, RPL21, RPL31, EIF1AY, RPS24, RPL34, RPS8, RPL30, RPL23, EIF3E, RPS29, RPL9, RPL27, RPS6, RPL26L1, RPS27L, RPL5, RPL6, RPS15A, RPS25, RPL41, MT-TM, EIF2AK2	<0.0001
Regulation of eIF4 and p70S6K Signaling		RPS3A, RPS27, ITGA2, RPS8, EIF3E, RPS29, RPS6, RPS7, RPS20, RPS27L, RPS25, RPS15A, EIF1AY, RPS24	<0.0001
Oxidative Phosphorylation		NDUFS5, ATP5J, NDUFA4, COX7B, COX6C, NDUFA6, COX7C, ATP5L, NDUFB3, NDUFS4, UQCRB	<0.0001
Mitochondrial Dysfunction		NDUFS5, NDUFA4, ATP5J, COX7B, COX6C, NDUFA6, COX7C, ATP5L, NDUFAF2, NDUFB3, UQCRB, NDUFS4, MT-ND6	<0.0001
mTOR Signaling		RPS3A, RPS27, RPS8, EIF3E, RPS29, RPS6, RPS7, RPS6KA6, RPS20, RPS27L, RPS25, RPS15A, RPS24, DIRAS3	<0.0001
**Ingenuity Disease or Function Annotation**		**Molecules**	***p*** ** Value**
Renal and Urological Disease	chronic kidney disease	AIF1, ATP5J, CA3, CLEC2B, COX6C, COX7C, DBI, EEF1B2, GABRB3, GSTM1, LSM3, NDUFA6, NDUFS5, PFDN5, RPL23, RPL34, RPL7, SNRPG, UQCRB, AVPR2, C3, CXCL12, SLC12A3	<0.0001
Cardiovascular Disease	intermediate disease stage peripheral arterial disease	CLEC2B, COX7C, EEF1B2, PFDN4, POLR2K, RPL9, SNRPG, SUB1, TOMM7, CXCL12	0.0020
	peripheral arterial disease	CLEC2B, COX7C, EEF1B2, PFDN4, POLR2K, RPL9, SNRPG, SUB1, THY1, TOMM7, CXCL12	0.0107
	peripheral vascular disease	CA3, CLEC2B, COX7C, EEF1B2, GABRB3, LY96, PDCD10, PFDN4, POLR2K, RPL9, S100A12, SNRPG, SUB1, THY1, TOMM7, CXCL12, KCNA5, SLC12A3	0.0123
Developmental Disorder	mitochondrial respiratory chain deficiency	NDUFAF2, NDUFB3, NDUFS4, UQCRB, MT-ND6, MT-TS1	0.0031
	Mitochondrial complex I deficiency	NDUFAF2, NDUFB3, NDUFS4, MT-ND6	0.0053
